# Supplementary material for: New Formulation towards Healthier Meat Products: Juniperus communis L. Essential Oil as Alternative for Sodium Nitrite in Dry Fermented Sausages
Source: Foods. 2020 Aug 6;9(8):1066. doi: 10.3390/foods9081066 (PMC7466274; doi:10.3390/foods9081066)
Supplement: Supplementary file 1 [file foods-09-01066-s001.pdf]

Table S1. Proximate chemical composition of dry fermented sausages.

FC (%)	moisture	protein	fat	ash
15	32.17±0.56	28.66±0.20	32.18±1.19	5.72±0.11
25	33.27±1.62	23.14±0.40	36.29±0.97	5.35±0.43

FC – fat; Means ± Stdev;

Table S2. The effect of two-way, three-way and four-way interactions among processing parameters on the quality of dry fermented sausages expressed as p-value

p	pH	*L**	*a**	*b**	TBARS	TPC	LAB	Hardness	Springiness	Cohesiveness	Chewiness	Color	Odor	Flavor
FC*NC	0.619	0.244	0.038	0.796	0.150	0.876	0.809	0.496	0.860	0.848	0.283	0.045	0.003	0.243
FC*SD	0.059	0.009	0.491	0.005	< 0.001	0.880	0.408	< 0.001	< 0.001	< 0.001	< 0.001	< 0.001	0.622	0.305
NC*SD	0.837	0.657	0.057	0.153	< 0.001	0.176	0.039	0.135	0.013	0.297	0.029	0.031	0.145	0.790
FC*JC	0.200	0.069	0.070	0.008	0.899	0.578	0.121	0.037	0.008	< 0.001	0.094	< 0.001	0.570	0.028
NC*JC	0.992	0.819	0.880	0.020	0.996	0.956	0.326	0.854	0.770	0.048	0.761	0.125	< 0.001	< 0.001
SD*JC	< 0.001	0.546	0.090	0.010	0.833	0.456	0.215	0.589	0.336	< 0.001	0.389	< 0.001	< 0.001	< 0.001
FC*NC*SD	0.005	0.626	0.003	0.001	< 0.001	0.805	0.990	0.118	< 0.001	0.645	< 0.001	< 0.001	0.719	0.841
FC*NC*JC	0.759	0.824	0.352	0.442	0.998	0.596	0.965	0.458	0.776	0.172	0.939	< 0.001	< 0.001	0.012
FC*SD*JC	0.001	0.167	0.030	0.395	0.002	0.656	0.658	0.002	0.006	0.142	< 0.001	< 0.001	0.096	< 0.001
NC*SD*JC	0.587	0.553	0.116	0.017	0.531	0.789	0.654	0.484	0.021	0.057	0.207	0.043	0.002	< 0.001
FC*NC*SD*JC	< 0.001	0.042	0.095	< 0.001	< 0.001	0.752	0.468	< 0.001	0.009	0.002	< 0.001	< 0.001	0.529	0.013

FC – fat content; NC – nitrite content; SD – storage day; JC – JEO content.
